# Surgical Wards

**Published:** 1871-12

**Authors:** 


### SURGICAL WARDS.

Service of PROF. ANDREWS.

CASE I. OPERATION TO REMEDY HAIR-LIP.—The patient, a little girl about two years old, is put under the influence ether, and a projecting tooth which is in the way is removed. The edges of the fissure having been pared are brought together by interrupted sutures; there is generally little difference in the result whether the interrupted sutures or the hair-lip pins be used, but when the front of the jaw is level the pins are rather better, and when there is a projection as in this case the interrupted sutures are preferable. There is always contraction of the cicatrix, and in order to avoid a notch being left at the lower end of the fissure after it is healed, the edges are cut a little concave so that the prolabium is projecting at present, and a suture is introduced through the prolabium also to prevent the production of the notch. The cut vessels are closed by torsion with unusual care because the child is to leave the city immediately after the operation, and therefore cannot be under observation. Finally a strip of adhesive plaster in the shape of an hour-glass is applied accross the lip and extending out on the cheeks, the parts having been previously drawn together; this prevents traction on the line of incision and the stretching apart of the healing edges.

CASE II. REMOVAL OF ENCYSTED TUMOR.—A little tumor apparently encysted, is seen on this patient's back over the ridge of the acromion; it is not very important but the patient is to be relieved of it. On cutting into it the white and nearly solid sebaceous contents are removed and the sac is cut out with the scissors, since it does not roll out easily as they usually do. Adhesive plaster 1½x6 inches is then put over the incision in lieu of stitches.

CASE III. ABSCESS IN THE RECTUM.—This man had an abscess at the side of the rectum, which was opened and the pus evacuated; but instead of healing in the ordinary way it became unhealthy, probably phagedenic. Now there is another fluctuating abscess on the other side of the rectum. It is difficult to see how there can be any syphilitic cause as the action is under the integument, but the unhealthy character of the abscesses gives the impression that some poison is there; accordingly after lancing the second fluctuating abscess they are both cauterized with nitric acid. Tincture of iron is prescribed, together with pure air and similar means of inducing plasticity.

---

CASE IV. URETHRAL STRICTURE.—After stricture of the urethra has been removed the canal frequently will tighten up again. The urethra under present examination was operated on some time ago in an eastern city and is now extraordinarily constricted. Yesterday a very small bougie was introduced and if this can be done again the man can be at once relieved; otherwise it is to be tried another day when there may be less resistance; there is also a false passage which acts as a pocket, and the instrument can be got past this only part of the time.

The surgeon succeeds in introducing a small filliform bougie which on account of its flexibility will follow the crooks and go where no metallic instrument can—on the end of this is a screw to connect with a ground steel staff, which is introduced with the bougie for a guide. A triangular blade, blunt in front, is then pushed down the groove of the staff and then withdrawn, the cutting edge dividing the stricture as the blade comes out. A catheter No. 10 afterwards goes into the bladder easily; it is to be passed daily, and, on leaving the Hospital, the patient must introduce it himself occcasionally for some time.

---

CASE V. Since the destruction of the Marine Hospital in the fire, the marines have been removed to Mercy Hospital.

This patient hurt his index finger while on board his vessel, and the articular ends of the phalanges are necrosed. Antiseptic dressings have been used, and the finger probably can be saved. The sailor prefers to have the finger, though a stiff one, rather than lose it. Thinking to save the finger by taking out the diseased bone, as in a larger limb, the necrosed bone is removed by the gouge and gouge-forceps. The wound is then dressed with carbolated cerate.

---

### Service of PROF. SHERMAN.

---

CASE I. Whenever a person is bit by a dog it is always understood that the dog was mad and the patient liable to have hydrophobia.

Susceptibility of the human system to hydrophobia, however, is now much doubted, the same termination occurring without any bite. The lecturer stated that he would not, himself, be so much troubled by a bite as by the cut of an old saw. But in the present condition of public opinion, immediate treatment is necessary. The wound is slight, but of course that is of little consequence if it has been inoculated.

There is a current notion among the people that to kill the dog cures the bite ; the affair being much like the events in the story of " The House that Jack Built." But *unfortunately* the dog has escaped and therefore some *other* remedy must be resorted to.

The wound will be strongly cauterized with nitric acid.

---

CASE II. FELON.—This patient has one of those deep inflammations under the fascia and tendons which are called felons, and are troublesome only from their locality. They may come in any portion of the body which is bound down tightly by fascia, and the pus which forms may dissect and burrow extensively. It is frequently thought best to delay opening a felon till it comes to a head fully ; but they should be opened early, especially if under the periosteum. A premature opening of the abscess has no bad results while delay may produce somewhat serious consequences.

---

Case III. This man's thumb has been jammed and the first joint broken into. Such an injury on a larger limb would require amputation, but the fingers seem to have greater conservative power than any other members. In a case which occurred lately a joint of the thumb was blown entirely off by a pistol ball, and to please the parents the bones were put in position and properly bandaged; no pus formed, and now the boy has a good thumb. The same experiment will be tried in the present instance. Of course amputation would give the speediest result, put the patient can wait, and if gangrene should ensue here, there would be no danger to the system. Many such cases are curable, which before the introduction of carbolic acid inevitably suppurated and were lost.

